# Clinical Limitations of Tissue Annexin A2 Level as a Predictor of Postoperative Overall Survival in Patients with Hepatocellular Carcinoma

**DOI:** 10.3390/jcm10184158

**Published:** 2021-09-15

**Authors:** Shu-Wei Huang, Yen-Chin Chen, Yang-Hsiang Lin, Chau-Ting Yeh

**Affiliations:** 1 Department of Gastroenterology and Hepatology, New Taipei Municipal Tucheng Hospital, New Taipei 236, Taiwan; huangshuwei@gmail.com; 2 Liver Research Center, Chang Gung Memorial Hospital, Linkou, Taoyuan 333, Taiwan; 3 Graduate Institute of Clinical Medicine, Chang Gung University, Taoyuan 333, Taiwan; sunnychen168@gmail.com

**Keywords:** hepatocellular carcinoma, annexin A2, prognostic marker, survival outcome

## Abstract

Hepatocellular carcinoma (HCC) is the second common cause of cancer-related death in Taiwan. Tumor recurrence is frequently observed in HCC patients receiving surgical resection, resulting in unsatisfactory overall survival (OS). Therefore, it is pivotal to identify effective prognostic makers, so that intensive surveillance or adjuvant treatments can be applied to predictively unfavorable patients. Previous studies indicated that Annexin A2 (ANXA2) was an effective prognostic marker in several cancers, including HCC. However, the prognostic value of ANXA2 in Taiwanese HCC patients remains unclear, where a great proportion of patients had chronic hepatitis B with liver cirrhosis. Here, ANXA2 was highly expressed in HCC tissues compared with para-neoplastic noncancerous tissues. Furthermore, high ANXA2 expression in HCC tissues independently predicted shorter OS. In subgroup analysis, however, ANXA2 expression could not effectively predict OS in the following subgroups: female, age > 65 years old, Child–Pugh classification B, hepatitis B virus surface antigen negative or anti-hepatitis C antibody positive, alcoholism, tumor number >1, presence of micro- or macrovascular invasion, absence of capsule, non-cirrhosis and high alpha-fetoprotein. In conclusion, ANXA2 expression in HCC tissues could predict postoperative OS. However, the predictive value was limited in patients with specific clinical conditions.

## 1. Introduction

Hepatocellular carcinoma (HCC) is the second most common cause of cancer-related death in Taiwan [1]. Infection with hepatitis B virus (HBV) and hepatitis C virus (HCV) can lead to chronic hepatitis, liver fibrosis, cirrhosis and eventually HCC [2]. Despite the improvement in the treatment of chronic viral hepatitis and the successful implantation of neonatal vaccination program against HBV, HCC is still a severe public health concern in Taiwan [3]. Surgical treatment is considered one of the most efficient therapies for early-stage HCC. However, incidence of tumor recurrence and distant metastasis remains high in HCC patients receiving surgical resection, resulting in unsatisfactory clinical outcomes. Several biomarkers such as alpha-fetoprotein (AFP) were used for diagnosis and outcome prediction in HCC patients. However, approximately 40% HCC patients still presented with normal levels of AFP, suggesting that the diagnostic and prognostic role of AFP in HCC patients is still limited [4,5]. Therefore, it is very important to identify new prognostic makers for these patients, so that more intensive surveillance and/or adjuvant treatments, if available, could be applied to unfavorable patients.

Annexin A2 (ANXA2) belongs to annexin family and is responsible for regulating cell growth, cell–cell junctions and apoptosis [6,7,8]. ANXA2 has been reported to act as an early-stage HCC biomarker [9]. Another study [10] reported that ANXA2 was overexpressed in hepatoma cells compared to normal cells. Depletion of ANXA2 repressed cell proliferation and enhanced 5-fluorouracil-mediated effects via suppression of β-catenin and cyclin D1 expression. Yang et al. [11] demonstrated that ANXA2 enhanced liver fibrosis through regulation of the von Willebrand factor (vWF) in vitro and in vivo. These findings suggest that ANXA2 plays an oncogenic role in HCC progression. However, another study [12] indicated that expression levels of ANXA2 in HCC tissue and serum specimens were not correlated well with clinical outcomes, suggesting that ANXA2 was not a good prognostic maker for HCC patients with HBV-related liver cirrhosis. Accordingly, the predictive value of ANXA2 in Taiwanese HCC patients needed to be determined, where a great proportion of HCC was HBV-related, arising from a cirrhotic background.

In this study, ANXA2 expression levels were determined by Western blot followed by densitometry-based quantification. The clinical correlation between ANXA2 expression and postoperative outcomes was analyzed in Taiwanese HCC patients.

## 2. Materials and Methods

### 2.1. Patients and Basic Clinical Data

This was a retrospective longitudinal cohort study. From 1996 to 2006, a total of 148 paired HCC specimens (cancerous and para-neoplastic noncancerous tissues) obtained from surgical resection of HCC in LinKou Chang Gung Memorial Hospital were retrieved (cohort 1) and subjected to ANXA2 expression analysis by Western blot. Samples providing sufficient amounts of protein for Western blot analysis were randomly selected from the tissue bank. Only those with written informed consent from patients were included. The clinicopathological data were collected, including age, gender, tumor number, tumor size, histological grading, microvascular invasion, macrovascular invasion, capsule, microsatellite distribution, liver cirrhosis, Child–Pugh classification of liver function, ascites, alpha-fetoprotein (AFP), albumin, bilirubin, prothrombin time (PT), aspartate transaminase (AST), alanine transaminase (ALT), HBV surface antigen (HBsAg), anti-HCV antibody and alcoholism (Table 1). Meanwhile, longitudinal data of recurrence-free survival (RFS) and overall survival (OS) were collected and calculated for survival outcome analysis. RFS was calculated as the period from the time of operation to the time of tumor recurrence or metastasis. OS was calculated as the period from the time of operation to the time of death. The time-point when a patient was lost to follow up was censored. In addition, expression levels of ANXA2 in online available datasets (TCGA, cohort 2 and GSE14520, (cohort 3) were analyzed to further confirm its prognostic value in patients with HCC [13].

### 2.2. Western Blot Analysis

The procedure of Western blot analysis was described in the previous study [14]. Cells were collected and lysed with RIPA buffer (BIOTOOLS Co., Ltd., Taipei, Taiwan, TAAR-ZBZ5) containing protease inhibitors (Merck Millipore, Temecula, CA, USA, #539134). Protein concentrations of these samples were determined using the Bradford assay. Protein samples (60 µg) were loaded and separated by SDS-PAGE. The voltage (V) at stacking gel and resolution gel was 60–80 and 120–150 V, respectively. After loading dye reached the end of the gel, the gel was transferred to 0.45 µm PVDF membrane. The blocking buffer was added to the membrane for 1 h at room temperature. The membrane was incubated with specific antibody against ANXA2 (BD Biosciences, Franklin Lakes, NJ, USA) overnight at 4 °C. In addition, β-actin (Sigma-Aldrich, St Louis, MO, USA) was also visualized and used as loading control. The signal intensity of ANXA2 and β-actin was calculated by Image Gauge software (Fujifilm, Tokyo, Japan).

### 2.3. Statistical Analysis

The univariate analysis, multivariate analysis, Kaplan–Meier survival curve and forest plot analysis were performed using SPSS version 20 (SPSS Inc., Chicago, IL, USA). *p* values < 0.05 were considered significant (* *p* < 0.05).

## 3. Results

### 3.1. Elevated ANXA2 Expression Is Negatively Correlated with Clinical Outcomes

**Figure 1:**
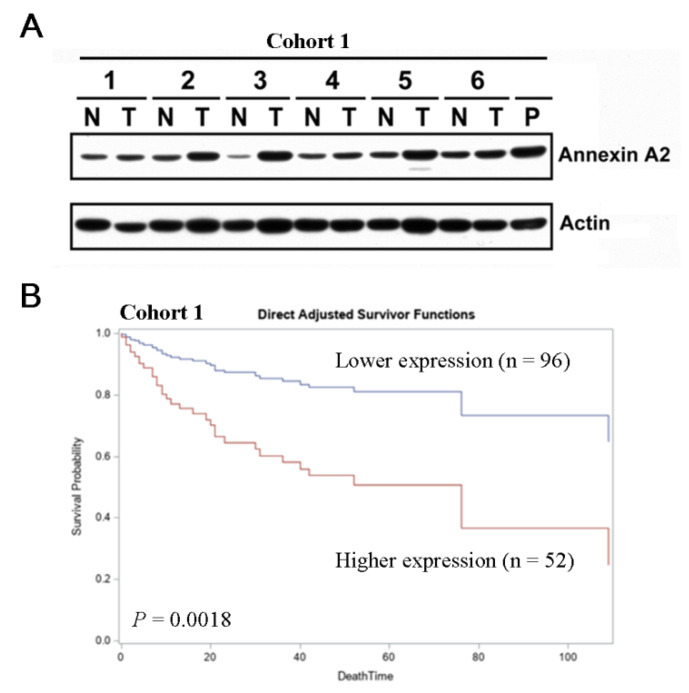
ANXA2 is clinically relevant in HCC. (**A**) Protein levels of ANXA2 in tumor tissues (T) and non-tumor tissues (N) were determined by Western blotting. *P*: positive control. (**B**) Kaplan–Meier survival curves with log-rank test stratified by high ANXA2 expression (higher T/N ratio ≥ 0.8) and low ANXA2 expression.

A total of 148 HCC patients receiving surgical resection were included. Of them, 80 (54%) patients were non-cirrhotic, and 68 (46%) of patients were cirrhotic. The basic clinical data were listed in Table 1. Compared with the non-cirrhosis group, the liver cirrhosis group had higher anti-HCV-positive rate, smaller tumor size, higher proportion of high ANXA2 expression, longer PT prolongation and lower AST level. To investigate whether ANXA2 acted as a prognostic biomarker, the expression levels of ANXA2 in HCC specimens were determined by Western blot followed by densitometry semi-quantification. The cancerous to non-cancerous (T/N) ratios of ANXA2 were calculated and the minimal *p* value method was applied to determine the cut off [15]. We found that ANXA2 expression was highly expressed in HCC tissues compared to noncancerous tissues (Figure 1A, *p* < 0.001). We retrieved the longitudinal data of RFS and OS to analyze whether AXNA2 expression (calculated as T/N ratio) was associated with prognosis in HCC. Kaplan–Meier plot with log-rank analysis showed that there was no significant association between AXNA2 expression (the T/N ratio) and RFS (*p* > 0.05). However, patients with high ANXA2 expression (T/N ratio ≥ 0.8) had a significantly shorter OS compared to those with low ANXA2 expression (Figure 1B). Similar results were observed in datasets available online (TCGA, cohort 2 and GSE14520, cohort 3) (Figure S1A,B). These findings clearly support that ANXA2 acts as a prognostic maker in patients with HCC. Notably, ANXA2 expression was positively correlated with cirrhosis, AST, anti-HCV antibody and the presence of capsule (Table 2). Taken together, ANXA2 might serve as a prognostic factor for HCC patients receiving surgical treatment.

### 3.2. Clinicopathological Predictors for RFS and OS

To identify the clinicopathological predictors for RFS and OS, univariate and multivariate Cox proportional hazard analysis was performed and is shown in Table 3 and Table 4. For RFS, presence of ascites, tumor number ≥ 2, presence of microvascular invasion and microsatellite distribution of tumors, high Annexin A2 expression, AFP and AST > upper limit of normal were associated with RFS by univariate analysis. Multivariate analysis showed that the presence of ascites, tumor number ≥ 2 and AST > upper limit of normal were the independent predictors for RFS (Table 3). For OS, age > 65 years, Child–Pugh liver function classification B, the presence of ascites, microvascular invasion, high ANXA2 expression, AFP, bilirubin and AST > upper limit of normal were associated with short OS in the univariate Cox proportional analysis. Multivariate analysis showed that Child–Pugh liver function classification B, presence of ascites and high AXNA2 expression were the independent predictors for OS (Table 4).

### 3.3. ANXA2 Expression Levels in HCC Tissues Are an Effective Prognosis Predictor in Specific Clinical Subgroups of HCC

**Figure 2:**
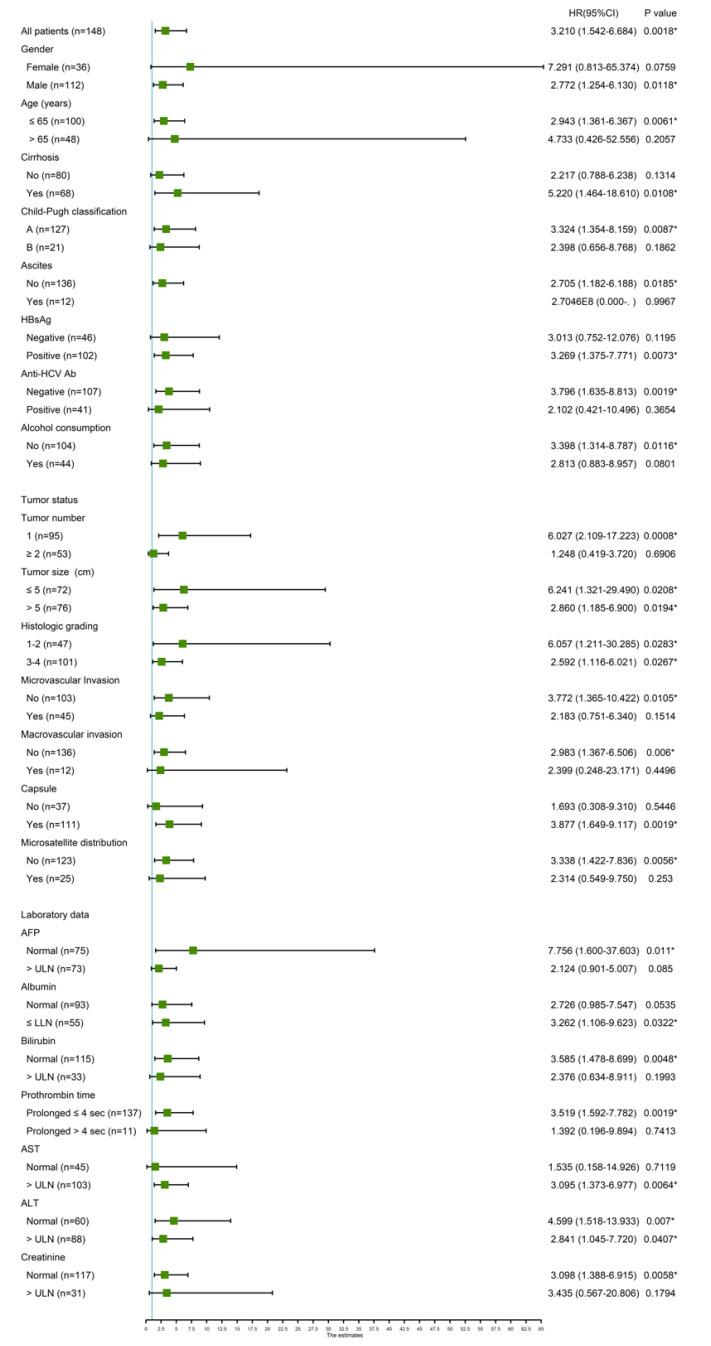
Forest plot of HRs for the associations between high AXNA2 expression and OS in various clinical subgroups. The subgroup-specific HR (95% CI) is shown by the green box (black lines). Statistically significant differences (*p* < 0.05) are indicated by a single asterisk ”*”.

In addition, we studied the predictive role of high AXNA2 expression in various clinical subgroups using Cox proportional hazard method (Figure 2). The ANXA2 expression was associated with OS when all HCC patients were included for assessment. In addition, it was also associated with OS in the following subgroups: male (HR = 2.772, 95% CI 1.254–6.130, *p* = 0.0118), age ≤ 65 (HR = 2.943, 95% CI 1.361–6.367, *p* = 0.0061), Child–Pugh liver function classification A (HR = 3.324, 95% CI 1.354–8.159, *p* = 0.0087), no ascites (HR = 2.705, 95% CI 1.182–6.188, *p* = 0.0185), HBsAg-positive (HR = 3.269, 95% CI 1.375–7.771, *p* = 0.0073), anti-HCV Ab negative (HR = 3.796, 95% CI 1.635–8.813, *p* = 0.0019), no alcohol consumption (HR = 3.398, 95% CI 1.314–8.787, *p* = 0.0116), tumor number =1 (HR = 6.027, 95% CI 2.109–17.223, *p* = 0.0008), tumor size ≤ 5 cm (HR = 6.241, 95% CI 1.321–29.490, *p* = 0.0208), tumor size > 5 cm (HR = 2.860, 95% CI 1.185–6.900, *p* = 0.0194), histological grading 1–2 (HR = 6.057, 95% CI 1.211–30.285, *p* = 0.0283) and 3–4 (HR = 2.592, 95% CI 1.116–6.021, *p* = 0.0267), no microvascular invasion (HR = 3.772, 95% CI 1.365–10.422, *p* = 0.0105), no macrovascular invasion (HR = 2.983, 95% CI 1.367–6.506, *p* = 0.006), presence of capsule (HR = 3.877, 95% CI 1.649–9.117, *p* = 0.0019), no microsatellite distribution (HR = 3.338, 95% CI 1.422–7.836, *p* = 0.0056), presence of cirrhosis (HR = 5.220, 95% CI 1.464–18.610, *p* = 0.0061), normal AFP (HR = 7.756, 95% CI 1.600–37.603, *p* = 0.011), Albumin ≤ LLN (HR = 3.262, 95% CI 1.106–9.623, *p* = 0.0322), normal bilirubin (HR = 3.585, 95% CI 1.478–8.699, *p* = 0.0048), Prothrombin time ≤ 4 s (HR = 3.519, 95% CI 1.592–7.782, *p* = 0.0019), AST > ULN (HR = 3.095, 95% CI 1.373–6.977, *p* = 0.0064), ALT normal (HR = 4.599, 95% CI 1.518–13.933, *p* = 0.007) and >ULN (HR = 2.841, 95% CI 1.045–7.720, *p* = 0.0407) and creatinine normal (HR = 3.098, 95% CI 1.388–6.915, *p* = 0.0058). In contrast, the association was not present in the following subgroups (*p* > 0.05 for all): female, age > 65 years, Child–Pugh classification B, presence of ascites, HBsAg negative, anti-HCV-positive, alcoholism; tumor number ≥ 2, micro- or macrovascular invasion, microsatellite distribution of tumors, non-cirrhosis, AFP or bilirubin > upper limit of normal, PT prolongation > 4 s, normal AST or creatinine > upper limit of normal.

Taken together, these findings suggest that high expression of ANXA2 in HCC cancerous parts could predict shorter OS in HCC patients receiving surgical treatment. However, in patients with more advanced stage of HCC or poorer liver function, non-cirrhosis patients or HBsAg-negative patients, the predictive value diminished.

## 4. Discussion

Previously, ANXA2 was identified as an independent prognostic marker in several cancer types, including laryngeal cancer [16], breast cancer [17], ovarian cancer [18] and endometrial cancer [19]. A similar predictive role of ANXA2 in HCC development has also been reported [9]. In the current study, we found that high expression of ANXA2 in HCC tissues was associated with a significantly shorter OS, indicating that ANXA2 was a predictor for unfavorable prognosis in liver cancer. Carbon tetrachloride (CCl_4_) treatment induces liver fibrosis, which mimics the sequel of chronic virus infection. Long-term CCl_4_ treatment renders fibrotic liver-to-liver cirrhosis, as a pre-malignant stage of HCC development [20]. Yang and co-workers demonstrated that ANXA2 levels were induced upon CCl_4_ treatment in Sprague Dawley rats compared to those in the control group [11]. Our results showed that ANXA2 expression was increased in patients with liver cirrhosis compared to those with non-cirrhosis (Table 2). Another report demonstrated that serum ANXA2 levels in chronic hepatitis B patients were significantly higher than those in the normal group [21]. On the other hand, a previous investigation indicated that ANXA2 functioned as a modulator in HCV assembly but not in HCV replication or viron release [22]. Our study revealed that ANXA2 expression was higher in the HCV-positive group compared to the HCV-negative group, suggesting ANXA2 expression was regulated by HCV infection through a yet unknown mechanism. However, in subgroup analysis, ANXA2 higher expression was not correlated with survival outcome in HCV-positive patients. Taken together, the evidence suggested that ANXA2 may be involved in early-stage HCC development, i.e., liver fibrosis to cirrhosis progression.

In contrast, Liu et al. indicated that ANXA2 expression in serum or HCC tissues were not significantly correlated with survival outcomes [12]. In an Egypt study, ANXA2 expression was lower in cirrhotic group than those in control group in HCC tissues [23]. These controversial results for ANXA2 on survival outcomes of HCC may be explained as follows: First, most of our specimens analyzed in this study were from cirrhotic or HBV-related patients. Second, in this study, ANXA2 expression in HCC tissues was detected by Western blot analysis followed by densitometry quantification. In contrast, an early study had assayed the serum levels of ANXA2 by ELISA. The detection method (Western blot vs. ELISA) and quantitative criteria may lead to different results. Third, these studies were performed in different countries; thereby, the geographic/ethnic issue may also have caused the inconsistent results. Fourth, a previous study reported that ANXA2 could be secreted to the extracellular environment upon interferon-γ treatment [24], suggesting that hepatitis activities might play a role. The intracellular and extracellular ANXA2 proteins also exert different functions [25]. We believe that these are possible reasons for the inconsistencies.

Zhang and co-workers demonstrated that knockdown of ANXA2 in hepatoma cell lines reduced cell migration and invasion [26]. Mechanistically, ANXA2 interacted with CD147 and regulated CD147 localization, thereby inducing matrix metallopoateinase 2 (MMP2) expression. Furthermore, knockdown of ANXA2 in a hepatoma cell line, MHCC97-H, repressed cell growth and invasive ability [27]. Oncogenic roles of transgelin-2 in HCC have been demonstrated, and its high expression is associated with ANXA2, which, in turn, promotes tumor metastasis through the NFκB pathway [28]. Another investigation indicated that ANXA2 was involved in immune escape of HCC via modulation of immune cells such as regulatory T cells, natural killer cells and dendritic cells [29]. In addition to HCC, the ANXA2-mediated immunosuppression phenotypes were observed in nasopharyngeal carcinoma cells [30] and renal cell carcinoma [31]. Another study revealed that expression levels of ANXA2 in liver tissues were upregulated in a thioacetamide (TAA)-induced cirrhotic rat model [32]. The authors found that immuno-related factors such as transforming growth factor beta and interleukin were increased in TAA-treated rats, suggesting ANXA2 might be involved in the immune response pathway. A long non-coding RNA, named lung cancer-associated transcript 1 (LUCAT1), induced cell growth and metastasis of hepatoma cell lines in vitro and in vivo [33]. LUCAT1 associated with ANXA2 was identified by an RNA pull-down assay, leading to inhibition of ANXA2 phosphorylation and induction of MMP9 activation. Recently, circular RNA (circRNA) has been found responsible for regulating cancer progression [34]. The expression levels of circ_0021093 were upregulated in HCC specimens, and a higher level of circ_0021093 was correlated with poor survival outcomes [35]. Depletion of circ_0021093 reduced cell proliferation, migration and invasion by modulating miR-432. Moreover, ANXA2 is a direct targeted gene of miR-432. These findings indicated that the circ_0021093/miR-432/ANXA2 axis was another important pathway regulating HCC progression. This evidence supported that ANXA2 played an oncogenic role in liver cancer.

## 5. Conclusions

In conclusion, we showed that ANXA2 was a prognostic marker for HCC patients receiving surgical treatment. However, the predictive value diminished in several clinical subgroups such as those with more advanced stage of HCC or poorer liver function, as well as non-cirrhosis and HBsAg-negative patients.

## Figures and Tables

**Table 1:** Basic clinicopathological factors of patients with or without liver cirrhosis (cohort 1).

Variable	All Patients	Non-Cirrhosis	Cirrhosis	*p*
Patient number	148	80	68	
Gender				
Female	36(24.3%)	20(25.0%)	16(23.5%)	0.835
Male	112(75.7%)	60(75.0%)	52(76.5%)	
Age (years)	56.0 ± 14.9	54.2±16.1	58.1 ± 13.2	0.106
Child–Pugh Classification				
A	127(85.8%)	70(87.5%)	57(83.8%)	0.523
B	21(14.2%)	10(12.5%)	11(16.2%)	
Ascites				
No	136(91.9%)	74(92.5%)	62(91.2%)	0.769
Yes	12(8.1%)	6(7.5%)	6(8.8%)	
HBsAg				
Negative	46(31.1%)	24(30.0%)	22(32.4%)	0.758
Positive	102(68.9%)	56(70.0%)	46(67.6%)	
Anti-HCV Ab				
Negative	107(72.3%)	68(85.0%)	39(57.4%)	<0.001 *
Positive	41(27.7%)	12(15.0%)	29(42.6%)	
Alcohol consumption				
No	104(70.3%)	59(73.8%)	45(66.2%)	0.315
Yes	44(29.7%)	21(26.3%)	23(33.8%)	
Tumor status				
Tumor number				
1	95(64.2%)	51(63.8%)	44(64.7%)	0.904
≥2	53(35.8%)	29(36.3%)	24(35.3%)	
Tumor size (cm)	6.9 ± 4.8	8.5 ± 5.1	5.1 ± 3.6	<0.001 *
Histological grading				
1–2	47(31.8%)	22(27.5%)	25(36.8%)	0.228
3–4	101(68.2%)	58(72.5%)	43(63.2%)	
Microvascular invasion				
No	103(69.6%)	54(67.5%)	49(72.1%)	0.548
Yes	45(30.4%)	26(32.5%)	19(27.9%)	
Macrovascular invasion				
No	136(91.9%)	74(92.5%)	62(91.2%)	0.769
Yes	12(8.1%)	6(7.5%)	6(8.8%)	
Capsule				
No	37(25.0%)	22(27.5%)	15(22.1%)	0.446
Yes	111(75.0%)	58(72.5%)	53(77.9%)	
Microsatellite distribution				
No	123(83.1%)	63(78.8%)	60(88.2%)	0.125
Yes	25(16.9%)	17(21.3%)	8(11.8%)	
Annexin A2 expression				
<0.8 (Low)	96(64.9%)	62(77.5%)	34(50.0%)	<0.001 *
≥0.8 (High)	52(35.1%)	18(22.5%)	34(50.0%)	
Laboratory data				
AFP (ng/mL)	14.9(1.5-327.500)	6.5(2.9–327.500)	22.0(1.5–89,637.7)	0.642
Albumin (g/dL)	3.7 ± 0.7	3.8 ± 0.7	3.7 ± 0.6	0.427
Bilirubin (mg/dL)	1.3 ± 1.7	1.4 ± 2.1	1.2 ± 1.0	0.550
Prothrombin time (sec)	12.4 ± 1.5	12.1 ± 1.5	12.7 ± 1.5	0.042 *
AST (U/L)	94.6 ± 118.8	111.7 ± 151.0	74.5 ± 57.7	0.044 *
ALT(U/L)	77.0 ± 96.8	87.9 ± 120.5	64.2 ± 56.0	0.119
Creatinine (mg/dL)	1.2 ± 1.4	1.3 ± 1.8	1.1 ± 0.7	0.428

Abbreviations: AST, aspartate aminotransferase; ALT, alanine aminotransferase; AFP, α-fetoprotein; HBsAg, hepatitis B surface antigen; Anti-HCV Ab, anti-hepatitis C virus antibody. * *p* < 0.05.

**Table 2:** Clinicopathological correlations of ANXA2 in HCC specimens (cohort 1).

Parameters	HCC Samples (*n* = 148)	Mean ± SE	*p* ^a^
Gender			
Male	112	0.6116 ± 0.0475	0.8186
Female	36	0.6583 ± 0.1143	
Age (years)			
≤65	100	0.6470 ± 0.0623	0.8222
>65	48	0.5729 ± 0.0518	
Cirrhosis			
No	80	0.4913 ± 0.0505	0.0003 *
Yes	68	0.7779 ± 0.0748	
Child–Pugh classification			
A	127	0.6213 ± 0.0505	0.6987
B	21	0.6333 ± 0.0942	
Ascites			
No	136	0.6191 ± 0.0483	0.4494
Yes	12	0.6667 ± 0.1157	
HBsAg			
Negative	46	0.7087 ± 0.0917	0.1533
Positive	102	0.5843 ± 0.0509	
Anti-HCV Ab			
Negative	107	0.5514 ± 0.0488	0.0039 *
Positive	41	0.8098 ± 0.0976	
Alcohol consumption			
No	104	0.6115 ± 0.0483	0.5390
Yes	44	0.6667 ± 0.1157	
Tumor number			
1	95	0.6316 ± 0.0579	0.8248
≥2	53	0.6500 ± 0.0730	
Tumor size			
≤5 cm	72	0.6528 ± 0.0544	0.2919
>5 cm	76	0.5947 ± 0.0826	
Histological grading			
1–2	47	0.6500 ± 0.0951	0.7070
3–4	101	0.6083 ± 0.0475	
Microvascular invasion			
No	103	0.6291 ± 0.0535	0.5899
Yes	45	0.6089 ± 0.0858	
Macrovascular invasion			
No	136	0.6154 ± 0.0482	0.2173
Yes	12	0.7083 ± 0.1202	
Capsule			
No	37	0.4378 ± 0.0647	0.0069 *
Yes	111	0.6847 ± 0.0553	
Microsatellite distribution			
No	123	0.6235 ± 0.0513	0.6822
Yes	25	0.6200 ± 0.0923	
AFP			
Normal	75	0.5533 ± 0.0414	0.6768
>ULN	73	0.6945 ± 0.0809	
Albumin			
Normal	93	0.6290 ± 0.0550	0.5360
≤LLN	55	0.6127 ± 0.0793	
Bilirubin			
Normal	115	0.6130 ± 0.0506	0.7281
>ULN	33	0.6575 ± 0.1017	
Prothrombin time			
Prolonged ≤ 4 s	137	0.6255 ± 0.0480	0.9589
Prolonged > 4 s	11	0.5909 ± 0.1254	
AST			
Normal	45	0.4311 ± 0.0490	0.0048 *
>ULN	103	0.7068 ± 0.0597	
ALT			
Normal	60	0.5800 ± 0.0742	0.2407
>ULN	88	0.6523 ± 0.0571	
Creatinine			
Normal	117	0.5820 ± 0.0424	0.3514
> ULN	31	0.7774 ± 0.1438	

^a^: Mann–Whitney U test (for two groups). * *p* < 0.05. Abbreviations: SE, standard error; ULN, upper limit of normal; LLN, lower limit of normal; AST, aspartate aminotransferase; ALT, alanine aminotransferase; AFP, α-fetoprotein; HBsAg, hepatitis B surface antigen; Anti-HCV Ab, anti-hepatitis C virus antibody.

**Table 3:** Analysis of factors that influenced RFS of all patients (cohort 1).

		RFS					
		Univariate Analysis			Multivariate Analysis		
Parameters	*n*	HR	95% CI	*p*	HR	95% CI	*p*
Gender							
Female	36						
Male	112	1.263	0.760–2.098	0.3668			
Age (years)							
≤65	100						
>65	48	0.785	0.491–1.256	0.3125			
Cirrhosis							
No	80						
Yes	68	1.466	0.967–2.223	0.0717			
Child–Pugh classification							
A	127						
B	21	1.345	0.714–2.535	0.3587			
Ascites							
No	136						
Yes	12	3.301	1.715–6.352	<0.001 *	2.274	1.156–4.472	0.0173 *
HBsAg							
Negative	46						
Positive	102	1.113	0.701–1.767	0.6507			
Anti-HCV Ab							
Negative	107						
Positive	41	1.180	0.736–1.891	0.4923			
Alcohol consumption							
No	104						
Yes	44	1.205	0.775–1.873	0.4069			
Tumor status							
Tumor number							
1	95						
≥2	53	3.240	2.097–5.005	<0.0001 *	2.649	1.571–4.467	0.0003 *
Tumor size (cm)							
≤5	72						
>5	76	1.475	0.968–2.247	0.0706			
Histological grading							
1–2	47						
3–4	101	1.217	0.760–1.950	0.4133			
Microvascular invasion							
No	103						
Yes	45	2.514	1.623–3.895	<0.0001 *	1.489	0.878–2.523	0.1394
Macrovascular invasion thrombosis							
No	136						
Yes	12	1.516	0.760–3.028	0.2379			
Capsule							
No	37						
Yes	111	0.778	0.486–1.245	0.2954			
Microsatellite distribution							
No	123						
Yes	25	2.300	1.391–3.803	0.0012 *	0.881	0.468–1.661	0.6962
Annexin A2 expression							
<0.8 (Low)	96						
≥0.8 (High)	52	1.726	1.120–2.659	0.0133 *	1.459	0.934–2.279	0.0969
Laboratory data							
AFP							
Normal	75						
>ULN	73	1.903	1.248–2.900	0.0028 *	1.544	0.994–2.399	0.0531
Albumin							
Normal	93	0.769	0.497–1.189	0.2369			
≤LLN	55						
Bilirubin							
Normal	115						
>ULN	33	1.384	0.847–2.261	0.1951			
Prothrombin time							
Prolonged ≤ 4 s	137						
Prolonged > 4 s	11	1.289	0.619–2.686	0.4981			
AST							
Normal	45						
>ULN	103	1.846	1.132–3.010	0.0141 *	1.719	1.027–2.880	0.0394 *
ALT							
Normal	60						
>ULN	88	1.304	0.852–1.995	0.2210			
Creatinine							
Normal	117						
>ULN	31	0.898	0.515–1.568	0.7057			

* *p* < 0.05. Abbreviations: RFS, recurrence-free survival; HR, hazard ratio; CI, confidence interval; ULN, upper limit of normal; LLN, lower limit of normal; AST, aspartate aminotransferase; ALT, alanine aminotransferase; AFP, α-fetoprotein; HBsAg, hepatitis B surface antigen; Anti-HCV Ab, anti-hepatitis C virus antibody.

**Table 4:** Analysis of factors that influenced OS of all patients (cohort 1).

		OS					
		Univariate Analysis			Multivariate Analysis		
Parameters	*n*	HR	95% CI	*p*	HR	95% CI	*p*
Gender							
Female	36						
Male	112	1.826	0.701–4.755	0.2175			
Age (years)							
≤65	100						
>65	48	0.345	0.121–0.986	0.0470 *	0.494	0.160–1.527	0.2204
Cirrhosis							
No	80						
Yes	68	1.357	0.677–2.720	0.3891			
Child–Pugh classification							
A	127						
B	21	4.894	2.345–10.215	<0.001 *	3.687	1.484–9.159	0.0050 *
Ascites							
No	136						
Yes	12	4.241	1.812–9.926	<0.001 *	3.361	1.328–8.507	0.0105 *
HBsAg							
Negative	46						
Positive	102	1.003	0.463–2.171	0.9949			
Anti-HCV Ab							
Negative	107						
Positive	41	1.026	0.460–2.287	0.9509			
Alcohol consumption							
No	104						
Yes	44	1.723	0.856–3.468	0.1275			
Tumor status							
Tumor number							
1	95						
≥2	53	1.666	0.807–3.439	0.1679			
Tumor size (cm)							
≤5	72						
>5	76	1.731	0.834–3.592	0.1410			
Histological grading							
1–2	47						
3–4	101	1.108	0.506–2.429	0.7974			
Microvascular Invasion							
No	103						
Yes	45	2.796	1.356–5.765	0.0053 *	1.921	0.873–4.227	0.1045
Macrovascular invasion thrombosis							
No	136						
Yes	12	2.488	0.953–6.495	0.0627			
Capsule							
No	37						
Yes	111	0.811	0.363–1.811	0.6097			
Microsatellite distribution							
No	123						
Yes	25	2.172	0.964–4.893	0.0612			
Annexin A2 expression							
<0.8 (Low)	96						
≥0.8 (High)	52	3.210	1.542–6.684	0.0018 *	2.497	1.109–5.619	0.0270 *
Laboratory data							
AFP							
Normal	75						
>ULN	73	2.292	1.102-4.766	0.0264 *	1.381	0.603–3.162	0.4446
Albumin							
Normal	93	0.515	0.257-1.036	0.0626			
≤LLN	55						
Bilirubin							
Normal	115						
>ULN	33	2.186	1.033-4.627	0.0410 *	1.077	0.457–2.538	0.8659
Prothrombin time							
Prolonged ≤ 4 s	137						
Prolonged > 4 s	11	2.031	0.773–5.340	0.1508			
AST							
Normal	45						
>ULN	103	3.362	1.179–9.586	0.0233 *	1.955	0.630–6.062	0.2458
ALT							
Normal	60						
>ULN	88	1.063	0.524-2.154	0.8662			
Creatinine							
Normal	117						
>ULN	31	0.683	0.263-1.776	0.4346			

* *p* < 0.05. Abbreviations: OS, overall survival; HR, hazard ratio; CI, confidence interval; ULN, upper limit of normal; LLN, lower limit of normal; AST, aspartate aminotransferase; ALT, alanine aminotransferase; AFP, α-fetoprotein; HBsAg, hepatitis B surface antigen; Anti-HCV Ab, anti-hepatitis C virus antibody.

## Data Availability

The available datasets can be analyzed and download from Gene Expression Profiling Interactive Analysis (http://gepia.cancer-pku.cn/, accessed on 1 September 2021, Beijing, China) and Gene Expression Omnibus (GEO; http://www.ncbi.nlm.nih.gov/geo, accessed on 1 September 2021, Bethesda MD, USA) with accession numbers GSE14520, respectively.

## Supplementary Materials

The following are available online at https://www.mdpi.com/article/10.3390/jcm10184158/s1, Figure S1: Elevated ANXA2 expression was correlated with poor prognosis in patients with HCC in online available dataset analysis.
