# Supplementary material for: Clinical Limitations of Tissue Annexin A2 Level as a Predictor of Postoperative Overall Survival in Patients with Hepatocellular Carcinoma
Source: J Clin Med. 2021 Sep 15;10(18):4158. doi: 10.3390/jcm10184158 (PMC8465313; doi:10.3390/jcm10184158)
Supplement: Supplementary file 1 [file jcm-10-04158-s001.zip › jcm-1358608-supplementary.pdf]

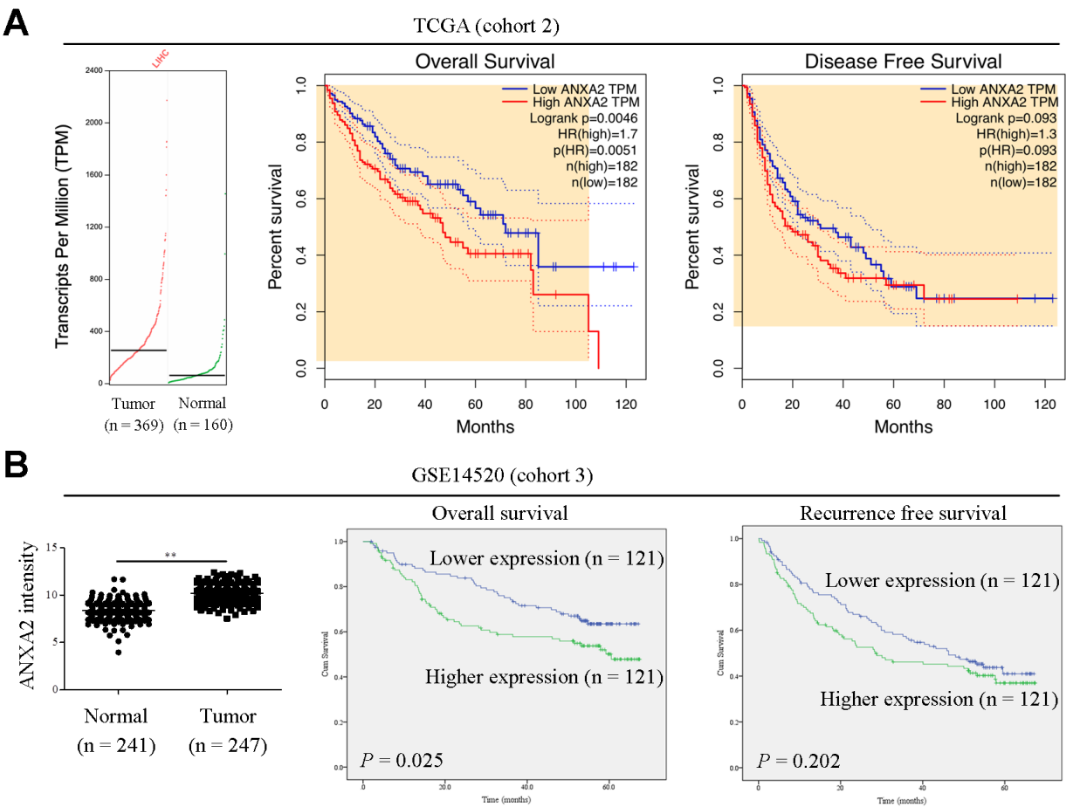


**Figure S1. Elevated ANXA2 expression was correlated with poor prognosis in patients with HCC in online available dataset analysis.**

(A and B) Levels of ANXA2 in two different cohorts (TCGA datbase, cohort 2 and GSE14520, cohort 3) were analyzed. Kaplan-Meier analysis of overall survival and recurrence free survival was performed with log-rank test based on ANXA2 expression. Median expression levels of ANXA2 were used as the cutoff.
